# Wharton's Jelly Mesenchymal Stem Cell Conditioned Medium Ameliorates Diabetes-Induced Testicular Damage and Sperm Abnormalities by Mitigating Oxidative Stress, Apoptosis, and Inflammation

**DOI:** 10.1155/2024/7084913

**Published:** 2024-10-03

**Authors:** Mojtaba Sargazi, Narges Karbalaei, Saied Karbalay-Doust, Sara Keshtgar, Zohre Aghaei

**Affiliations:** ^1^Department of Physiology, School of Medicine, Shiraz University of Medical Sciences, Shiraz, Iran; ^2^Histomorphometry and Stereology Research Center, Shiraz University of Medical Sciences, Shiraz, Iran; ^3^Department of Anatomy, School of Medicine, Shiraz University of Medical Sciences, Shiraz, Iran

## Abstract

Diabetes leads to testicular damage and infertility. Mesenchymal stem cells and their secretory trophic factors have shown potential as regenerative therapies for diabetes and its associated complications. This study examined the effects of conditioned medium derived from Wharton's jelly mesenchymal stem cells (WJMSCs-CM) on sperm parameters, reproductive hormones, biochemical parameters, and histological changes in the testes of diabetic rats. Fifty-six male Sprague–Dawley rats (250–300 g) were assigned to eight groups: control, diabetes, and six diabetic groups receiving early or late treatments with WJMSCs-CM (D-CM_E_, D-CM_L_), insulin (D-INS_E_, D-INS_L_), or DMEM (D-DM_E_, D-DM_L_). In the early treatment groups, insulin (3 U/day, subcutaneously) and WJMSCs-CM (10 mg/week, intraperitoneally) were administered immediately after diabetes induction; in the late treatment groups, these interventions began 30 days postinduction. Blood glucose and insulin levels, along with sperm parameters, were assessed. Sex hormones, testicular antioxidant enzyme activity, malondialdehyde (MDA), and glutathione (GSH) concentrations were measured using colorimetric methods. Real-time PCR detected Bax, Bcl-2, and tumor necrosis factor-alpha (TNF-*α*) gene expression. Our results showed that diabetes increased blood glucose levels, decreased insulin and sex hormone levels, induced testicular oxidative stress and apoptosis, and reduced sperm parameters compared to the control. WJMSCs-CM significantly ameliorated hyperglycemia, increased insulin and sex hormone levels, and improved sperm quality. In WJMSCs-CM-treated diabetic rats, MDA levels were reduced, while GSH and antioxidant enzyme activity increased. Furthermore, WJMSCs-CM decreased the testicular Bax/Bcl-2 ratio and TNF-*α* expression, as well as enhanced spermatogenic, Sertoli, and Leydig cells. In conclusion, WJMSC-CM administration effectively mitigated diabetes-induced testicular damage by reducing oxidative stress, inflammation, and apoptosis. Early treatment with WJMSCs-CM was more effective than late treatment for diabetes-induced reproductive dysfunction.

## 1. Introduction

Diabetes mellitus is a prevalent chronic metabolic disorder that occurs worldwide. It is distinguished by hyperglycemia due to insufficient insulin production and/or insulin resistance. Chronic hyperglycemia has detrimental effects on various organs, resulting in problems such as neuropathy, cardiovascular dysfunction, nephropathy, and blindness. Additionally, it has important implications for male reproductive health [1, 2]. Diabetes is associated with an increased risk of reproductive problems in both humans and animals [3]. A few of its effects include structural and functional abnormalities in the testis and disturbances to the hypothalamic–pituitary–gonadal (HPG) axis, which leads to low levels of sex hormones like gonadotropin-releasing hormone (GnRH), follicle-stimulating hormone (FSH), luteinizing hormone (LH), and testosterone, along with structural and functional abnormalities in the testis [2]. Testicular atrophy, seminiferous tubule disruption, and decreased spermatogenesis are all symptoms of diabetes-induced male reproductive dysfunction, which can result in infertility [2, 4]. Multiple clinical trials have also reported erectile and ejaculatory dysfunction, decreased semen volume, low sperm quality, and abnormal sperm morphology [5, 6]. One of the main reasons for these reproductive problems is oxidative stress that sperm experiences due to prolonged hyperglycemia. Prolonged hyperglycemia in diabetes increases reactive oxygen species (ROS) production and decreases antioxidant efficacy [2, 7, 8]. ROS induces the overexpression of genes involved in testicular inflammation, affecting cell membrane and DNA integrity [9, 10]. Conventional treatments for diabetes, while partially effective, often come with side effects and may not fully mitigate the risk of complications [11, 12, 13]. Mesenchymal stem cell (MSC) therapy, or the use of bioactive substances released by these cells, is one topic that has gained attention in recent years as a potential treatment for diabetes and its related problems.

MSCs are increasingly recognized as promising options for treating several disorders due to their regenerative properties and ability to secrete bioactive substances [14, 15]. Recently, there has been consideration of using MSCs or the bioactive chemicals produced by these cells as a therapy for diabetes and its associated effects. The secretomes of MSCs contain bioactive substances such as growth factors, cytokines, extracellular proteins, and exosomes. MSCs-derived secretomes can facilitate tissue repair and regeneration through paracrine activities [14, 16]. Conditioned medium obtained from MSCs (MSCs-CM) is a cell-free substance composed of MSCs released products. Because MSCs-CM has similar effects to MSCs, it can be used as a viable alternative treatment [17]. MSCs-CM has exhibited various biological properties such as tissue regenerating, antiapoptotic, antioxidant, and anti-inflammatory effects [18, 19, 20]. Multiple studies have shown the beneficial impact of MSC_S_-CM on various disorders [18, 21, 22].

This research used Wharton's jelly stem cells (WJMSCs) from the HUC to prepare MSCs-CM, which have advantages such as rapid proliferation, lower immunological rejection risk, and abundant collection sources [17, 23, 24]. Several animal studies and clinical trials have tested the efficacy of WJMSCs or their secretory bioactive factors in various diseases, including neurological disorders, cardiac disease, immunological diseases, cancer, liver disease, bone/cartilage disease, and diabetes and its complications [15, 17, 25, 26].

In this study, we aimed to investigate whether the substances released from WJMSCs in the culture medium (WJMSCs-CM) could have therapeutic effects on male reproductive disorders caused by diabetes. Specifically, our focus was on assessing the impact of WJMSCs-CM on sperm parameters, inflammatory markers, oxidative stress, and histological alterations in the testes of diabetic rats.

## 2. Materials and Methods

### 2.1. Ethical Approval

All animal experimentation procedures were assessed and approved by the Animal Care and Local Ethics Committee of Shiraz University of Medical Sciences (IR.SUMS.AEC.1401.045).

### 2.2. Animals and Experimental Design

The 12 weeks adult male Sprague–Dawley rats weighing 250–300 g (*n* = 56) were purchased from the Center of Comparative and Experimental Medicine of Shiraz University of Medical Sciences and used in this study. The rats were housed in a controlled laboratory environment with a 12-hr cycle of darkness and light, temperature 23 ± 2°C, humidity 35%–40%, and unrestricted access to normal rat food and water throughout the experiment.

Animals were given 2 weeks for acclimatization before any experiments began. Next, the rats were assigned at random to control (*n* = 7) and streptozotocin (STZ)-induced diabetic groups (*n* = 49). After diabetes induction with an intraperitoneal injection of STZ, the diabetic group was further randomly subdivided into an untreated diabetic group and early- and late-treated diabetic groups. In the early treatment groups, therapy was initiated immediately after the induction of diabetes and continued for 8 weeks, with diabetic rats receiving WJMSCs-CM (D-CM_E_), insulin (D-INS_E_), and DMEM, Dulbecco's modified eagle medium (D-DM_E_). In the late diabetic groups, treatment with WJMSCs-CM (D-CM_L_), insulin (D-INS_L_), and DMEM (D-DML) began 30 days after the induction of diabetes and lasted for 4 weeks. In the treated diabetic groups, insulin (3 U/day) [18] was administered subcutaneously, while WJMSCs-CM (10 mg/week) and DMEM (10 mg/week) were given intraperitoneally [18].

### 2.3. Diabetic Induction

To induce diabetes, fasted (12 hr) rats were injected intraperitoneally with STZ (55 mg/kg; Solarbio Life Science) freshly dissolved in 0.1 M citrate buffer (pH 4.5). Seven days after STZ injection, blood glucose concentration was measured using a glucometer (Arkray, Japan), and animals with blood glucose levels above 250 mg/dL were accepted as diabetic and included in this study.

### 2.4. Procedure for Extracting and Culturing WJMSCs from the Umbilical Cord

Removed human umbilical cord (HUC) samples were randomly collected from healthy full-term neonates born at Shiraz University Hospital following institutional board permission and written informed consent. Within 4–24 hr of birth, umbilical cords were transported to our laboratory. After three rinses in phosphate-buffered saline (PBS) and vessel separation, the delicate Wharton's jelly tissue was extracted from the amniotic layer and chopped into small pieces. The WJMSCs were extracted by cutting the tissue and digesting it with collagenase and hyaluronidase enzymes. The separated cells were subsequently grown in T-25 culture flasks using Dulbecco's modified eagle medium (DMEM) supplemented with 15% fetal bovine serum and 1% penicillin/streptomycin (P/S) at 37°C, with media change occurring twice a week [18]. In our colleague's study, such cells were identified as MSCs by recognizing specific cell surface markers on these cells [27].

### 2.5. Preparation of WJMSCs Conditioned Medium (CM)

The study used WJMSCs from the fourth passage for CM collection. After 80% confluence, the supernatant was removed, and cells were washed with PBS. The cells were cultured in serum-free DMEM for 48 hr. The resulting CM was centrifuged to remove debris and filtered [18].

The supernatants were processed into a lyophilized powder in a freeze dryer (Christ Alpha1-2 LD Plus, Germany), concentrated 50 times, and quantified using the bicinchoninic acid (BCA) Protein Assay Kit (Rockford, IL) and for later use stored at −80°C. After preparing lyophilized CM powder, it was dissolved in distilled water and injected intraperitoneally at a concentration of 10 mg/mL for each rat once a week [28].

### 2.6. Sample Collection

At the end of the experiment, the animals were deeply anesthetized with a high dose of ketamine–xylazine (300/30 mg/kg), and blood samples were collected through cardiac puncture. After centrifuging of blood (3,000 g for 10 min), serum samples were collected and stored at –20°C for later measurements of serum glucose and hormone levels. After that, rats were euthanized by decapitation under deep anesthesia and testes were immediately removed and weighed. The left testis was stored at –80°C for real-time PCR investigation and oxidative stress evaluation, and the right testis was fixed in a 10% neutral buffered formalin solution for histological study.

### 2.7. Serum Glucose and Hormonal Analysis

The serum level of glucose was assessed by the glucose oxidase procedure (Pars Azmoon Co., Tehran, Iran). Serum concentrations of FSH, LH, and total testosterone were measured using the ELISA method following the manufacturer's instructions for commercial kits (Cusabio Biotech Company, Wuhan, China). The serum insulin levels were determined using a rat ELISA kit (Insulin, Mercodia, Uppsala, Sweden). All measurements were performed in duplicate.

### 2.8. Body and Testis Weight, Gonadosomatic Index

During the experiment, the body was recorded once a week. The testis weight and gonadosomatic index of each animal were measured. The gonadosomatic index was determined by multiplying the ratio of testicular weight to body weight by 100 and expressing it as a percentage.

### 2.9. Sperm Collection

To maintain the sample's integrity and quality, sperm collection was done under deep anesthesia before cardiac puncture. For the collection of viable sperm samples, the tail of epididymis was dissected and rapidly put in a small petri dish counting 5 mL PBS, and incubated at 37°C for 30 s to diffuse the semen. The semen solution was quietly vibrated to obtain uniform seminal fluid.

### 2.10. Sperm Analysis

#### 2.10.1. Sperm Count

The seminal fluid was spread on a Neubauer hemocytometer and the sperm heads were counted manually by light microscope. About 200–300 head of sperms were counted in every rat and results were expressed as total sperm count per milliliter [29, 30].

#### 2.10.2. Sperm Motility

The seminal fluid was loaded in preheated microscope slides. Ten microscopic fields at a final magnification of 400x randomly selected and 200–300 sperms per animal were evaluated. Sperm motility was classified as progressive motile, nonprogressive, and nonmotile according to the fifth edition of the World Health Organization guidelines [31]. The percentage of sperm motility was assessed by number of motile sperm × 100/total number of spermatozoa [29, 30].

#### 2.10.3. Sperm Viability

For sperm viability, the smear of seminal fluid was stained with eosin and nigrosin dyes (Sigma–Aldrich) according to the World Health Organization guidelines. The heads of living sperms are not dyed, but the heads of dead sperms are dyed. The 200–300 stained and nonstained sperms per rat at a final magnification of ×400 in 10 random microscopic fields were evaluated. The percentage of live sperm was assessed by the number of live sperm × 100/sum of live and dead sperm [29, 30].

#### 2.10.4. Sperm Morphology

To assess sperm morphology, 40 *μ*L sperm suspension was gently mixed with 10 *μ*L solution containing 1% eosin *Y* and nigrosin in a test tube. The sperm was incubated at room temperature for 60 min to facilitate staining. Subsequently, the sperm was resuspended using a pipette. A light microscope examined 200 spermatozoa on each slide (magnification: 400x). The abnormalities were classified as head and tail defects (double head, flattened head, bent tail, curved neck). The percentages of normal sperms were determined [32].

### 2.11. Evaluation of Oxidative Stress Markers

For assessment of the testicular oxidative stress parameters, 100 mg of the testis was weighed, then cut into small pieces and homogenized in cold PBS (pH = 7.4). Supernatants were isolated by centrifugation with 1 mM EDTA at 4,000 rpm for 15 min at 4°C and their supernatants were separated and used for lipid peroxidation, enzymatic activity, and protein quantitation studies. Malondialdehyde (MDA) content as a lipid peroxidation index was measured using the thiobarbituric acid reactive substance method [8]. Superoxide dismutase (SOD), glutathione peroxidase (GPx), and catalase (CAT) activities, as well as glutathione concentration (GSH), were quantified with commercial test kits (Kiazist Life Sciences, Iran) using colorimetric methods. A BCA protein quantification kit (Pars Tous Biotech, Iran) was used to quantify the amount of protein in the tissues.

### 2.12. Total RNA Extraction and Real-Time PCR

First, the testis samples were sonicated with the help of ultrasound waves for 5 min with 100% power of the device on ice and in PBS solution. Then, the samples were centrifuged at 2,500 rpm and the supernatant was removed for the next step. The trizol/chloroform column extraction method was used to extract RNA from testis samples. For this purpose, Pars Tous company's RNA extraction kit was used. Then, using a cDNA Synthesis Kit (Fermentas et al.), the whole RNAs were reverse transcribed into cDNA according to the manufacturer's instructions.

After that, the testicular expression of B-cell lymphoma 2 (Bcl-2), Bcl-2-associated X protein (Bax), and tumor necrosis factor-alpha (TNF-*ɑ*) genes were conducted by real-time PCR on StepOne real-time PCR system using SYBR Green High ROX Master Mix (Amplicon, Brighton, UK) according to manufacturer's protocol. Glyceraldehyde-3-phosphate dehydrogenase (GAPDH) was considered as an internal control. The relative expression level of genes was calculated by the 2^−*ΔΔ*Ct^ formula. The sequences of primers used in this study are as follows:

For: Bax, F: 5′-GCTACAGGGTTTCATCCAGG-3′; R: 5′-TTGTTGTCCAGTTCATCGCC-3′;

For: Bcl2, F: 5′-CTGGTGGACAACATCGCTCT-3′; R: 5′-GCATGCTGGGGCCATATAGT-3′;

For: TNF-*ɑ*, F: 5'-TCAGCCTCTTCTCATTCCTGC-3′; R: 5'-TTGGTGGTTTGCTACGACGTG-3′;

For: GAPDH, F: 5′-AGTGCCAGCCTCGTCTCATA-3′; R: 5′-GAGAAGGCAGCCCTGGTAAC-3′.

### 2.13. Stereological Studies

#### 2.13.1. Testis Tissue Collection and Preparation

**Figure 1:**
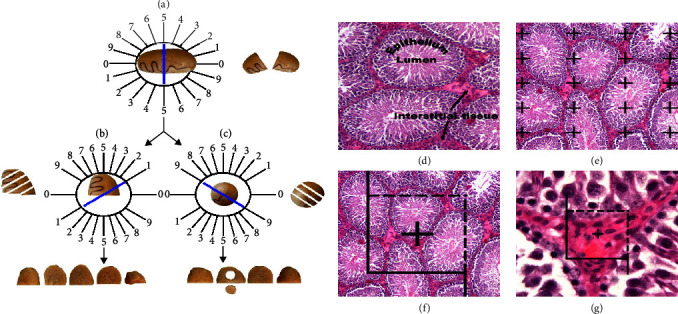
Application of stereological techniques: isotropic uniform random sections of testicular tissue were obtained using the random direction of the *Φ* and *θ* o'clock (a–c). The testis is located on *Φ* o'clock and a random number (here 5), the testis was divided into two parts (a). Then, the cutting surface of each part of the testicle is located in a 0–0 direction and the second cuts are made (here 1 and 9) (b and c). As a result, each testicle was cut in the form of parallel plates with the second cutting direction with a distance of 1 mm, and 8–12 slabs were prepared (b and c). The testicular tissue (seminiferous tubules, seminiferous epithelium, lumen, and interstitial tissue) is demonstrated in the histological sections (d). The point-counting technique was used to assess the volume density of the testicular tissue on the hematoxylin and eosin (H&E) stained tissue sections (e). An unbiased counting frame was employed to assess the seminiferous tubule length density (f). The disector method was employed for estimating the numerical density of the testicular tissue cells (g).

After inducing deep anesthesia and sacrificing the rats, their testes were weighted, and primary testicular volume was measured with the immersion method in normal saline. To evaluate the stereological parameters such as tubule length, isotropic uniform random sections should be performed. So, the testis was cut according to the “orientator method” (Figures 1(a), 1(b), and 1(c)). The segments were maintained in 10% neutral buffered formalin for tissue processing. Afterward, the segments (8–12) were processed, embedded into blocks of paraffin, sectioned (5 and 25 *µ*m thickness), and stained with Hematoxylin and Eosin (H&E). After that, 8–12 fields were selected from each microscopic slide according to systematic uniform random sampling, and stereological parameters were calculated [33].

Usually, tissue processing results in a shrinkage of the testis. A simple way to evaluate the degree of shrinkage “d(shr)” is to punch a circular piece from the sections of the testis with a trocar (Figure 1(c)). Afterward, the areas of the circle after and before tissue processing were measured.

The degree of testicular shrinkage “d(shr)” was calculated by the subsequent formula:

(1) $$\begin{array}{c} d\left(\text{shr}\right)=1-{\left(\text{ AA}/\text{AB }\right)}^{1.5}, \end{array}$$

where AA and AB are the circular parts of the testis after and before tissue processing, respectively. The secondary volume of the testis was estimated by multiplying the primary volume and the degree of shrinkage [33].

#### 2.13.2. Estimation of the Seminiferous Tubules, Epithelium, and Interstitial Tissue Volumes

Five micrometers thick sections were used to estimate the volume of the seminiferous tubules, seminiferous epithelium, and interstitial tissue. The stereology tool was prepared from a Nikon E-200 microscope (Tokyo, Japan) with an oil objective lens (Plan Apo 60x, n/a: 1.4, Tokyo, Japan) and a Samsung video camera (SCB-2000P, Hanwha Techwin, South Korea) connected to a computer. The stereological probe was employed for the live image and the mentioned parameters were estimated with the “point-counting way,” with a stereology software system (Stereolite, SUMS, Shiraz, Iran) [33]. The volume density “*Vv* (structure/testis sections)” of the testis was assessed by the “point-counting method” (Figures 1(d) and 1(e)) and the after that formula:

(2) $$\begin{array}{c} V\left(\text{structure}\right)=\left[\sum P\left(\text{structure}\right)/\sum P\left(\text{total}\right)\right]\times V\left(\text{secondary volume testis}\right), \end{array}$$

where ∑*P* (structure) and ∑*P* (total) are the number of points that hit the seminiferous tubules, seminiferous epithelium, interstitial tissue, and the entire testicular tissue, respectively.

#### 2.13.3. Estimation of Seminiferous Tubule Length

Five micrometers thick sections were used to evaluate the length density of seminiferous tubules with an unbiased counting frame. The unbiased counting frame has two prohibiting lines (the left and inferior sides and their extensions), and two acceptable lines (the right and superior sides) were superimposed on the image [33]. The seminiferous tubules are counted either completely or partially inside the unbiased counting frame and do not touch the outlet lines.

The length density (*L*_V_) of the seminiferous tubules is assessed by the next formula:

(3) $$\begin{array}{c} L_{\text{V}}\left(\text{seminiferous tubules}\right)=2\sum Q/\left(\sum P\times a/f\right), \end{array}$$

where ∑*Q* is the sum of counted seminiferous tubules, ∑*P* is the total points at the center of the unbiased counting frame that strikes the testicular tissue, and *a*/*f* is the area per counting frame [33] (Figure 1(f)). The total length of the seminiferous tubules was estimated by multiplying the length density and the secondary volume of the testis.

#### 2.13.4. Stereological Estimation of the Cell Number

The numerical density “*N*_V_” of spermatogonia, spermatocytes, spermatids, Sertoli, and Leydig cells was estimated using the optical disector method on 25 *µ*m thick sections.

The optical disector setting consisted of an Eclipse microscope (E200, Nikon, Tokyo, Japan) with a 40X oil immersion objective lens (Nikon, Japan, with a high numerical aperture (NA = 1.3)) which was connected to a video camera, that sent live microscopic image to a monitor and an electronic microcator (MT12, Heidenhain, Traunreut, Germany), for evaluating the movements in the *Z* direction.

An unbiased counting frame is used to count the number of spermatogenic, Sertoli, and Leydig cells that were explained. The guard's zones are above and below the surfaces of the histological sections, which are employed to prevent artifacts that occur during tissue processing in these sections.

The cells inside the guard's zones were not counted. The distance between the upper and lower guard's zones was equal to the height of the disector. All cells were identified, and each cell with a high level of focus in the frame, and completely or partially inside the counting frame without hitting the prohibited lines, was counted (Figure 1(g)).

The numerical density (*N*_V_) of the spermatogenic, Sertoli, and Leydig cells was estimated with the following formula:

(4) $$\begin{array}{c} N_{\text{V}}\left(\text{cells}/\text{testis}\right)=\sum Q/\left[\sum P\times a\left(f\right)\times h\right], \end{array}$$

where *ΣQ*^−^ is the total number of counted cells within the sampling volume, *ΣP* is the total of counted frames in the entire microscopic fields, *a*(*f*) is the area of the frame, and *h* is the height of disector. The total number of cells was calculated by multiplying the numerical density (*N*_V_) and the secondary volume of the testis.

### 2.14. Statistical Analysis

The data are analyzed using GraphPad Prism (Version 8.4.3). The normality of the data was assessed using the Kolmogorov–Smirnov test (K–S test). All data except the BAX, Bcl2, and Bax/ Bcl2 variables had a normal distribution, and their comparisons were conducted using one-way ANOVA followed by the Tukey HSD test. The BAX, Bcl2, and Bax/Bcl2 variables were statistically analyzed with the Kruskal–Wallis nonparametric test. Parametric data are expressed as mean ± SEM; nonparametric data were reported as median with IQR. *P* < 0.05 is considered the level of significance for all analyses.

## 3. Results

### 3.1. Serum Glucose and Insulin Levels

**Figure 2:**
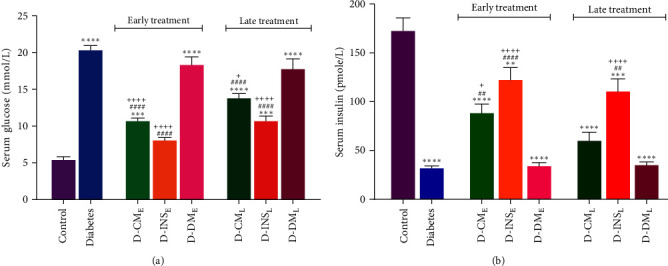
Serum levels of glucose (a) and insulin (b) in the control, diabetes, early and late insulin, CM, and DMEM-treated diabetic groups. Data were analyzed by one-way ANOVA (Tukey's HSD test) are presented as mean ± SEM (*n* = 7).  ^*∗*^ ^*∗*^*P* < 0.01,  ^*∗*^ ^*∗*^ ^*∗*^*P* < 0.001,  ^*∗*^ ^*∗*^ ^*∗*^ ^*∗*^*P* < 0.0001 vs. the control group; ^##^*P* < 0.01, ^####^*P* < 0.001 vs. the diabetic group; and ^**+**^*P* < 0.05, ^++++^*P* < 0.0001 vs. the D-DM_E_ and D-DM_L_. D-CM_E_, diabetic group receiving early WJMSCs-CM; D-CM_L_, diabetic group receiving late WJMSCs-CM; D-INS_E_, diabetic group receiving early insulin; D-INS_L_, diabetic group receiving late insulin; D-DM_E_, diabetic group receiving early DMEM; D-DM_L_, diabetic group receiving late DMEM; early, treatment was started immediately after diabetes induction; late, treatment was started 30 days after diabetes induction.

Our findings showed that diabetic rats present high serum glucose and low serum insulin levels compared with control rats. Early and late treatments with WJMSCs-CM and insulin resulted in a statistically significant decrease in serum glucose compared to diabetic rats. Insulin levels in three groups of D-INS_E_, D-CM_E_, and D-INS_L_ were higher than those in the diabetic group. However, the serum glucose and insulin levels in early and late WJMSCs-CM-treated diabetic groups did not reach the control group. Serum insulin levels in D-INS_E_ and D-INS_L_ were significantly lower, and serum glucose levels in D-INS_L_ were considerably higher than the control group (Figures 2(a) and 2(b)). Early and late administration of DMEM did not alter serum glucose and insulin levels compared to the diabetes group. The serum glucose level in early- and late-treated groups with WJMSCs-CM and insulin was lower than in D-DM_E_. Serum insulin in two groups of D-CM_E_ and D-INS_E_ was higher than D-DM_E_. Late treatment with insulin showed a difference in insulin levels with D-DM_L_. There were no differences in serum glucose and insulin levels between late and early administration insulin and CM.

### 3.2. Body Weight Gain, Testis Weight, and Gonadosomatic Index

**Figure 3:**
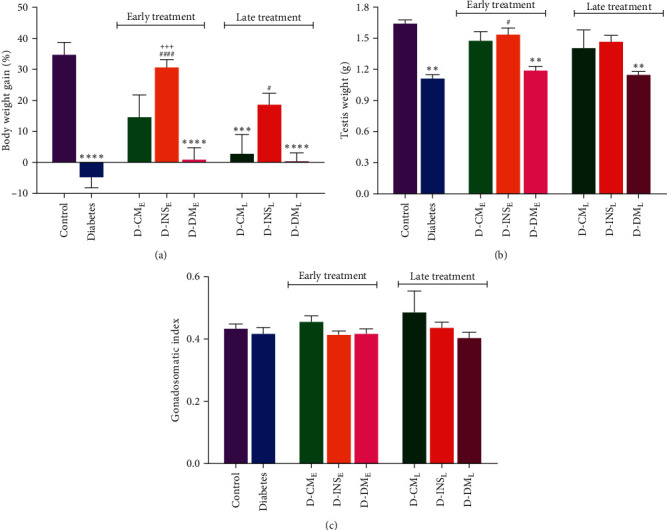
Body weight gain (a), testis weight (b), gonadosomatic index (c) in the control, diabetes, early and late insulin, CM, and DMEM-treated diabetic groups. Data were analyzed by one-way ANOVA (Tukey's HSD test) and presented as mean ± SEM (*n* = 7).  ^*∗*^ ^*∗*^*P* < 0.01,  ^*∗*^ ^*∗*^ ^*∗*^*P* < 0.001,  ^*∗*^ ^*∗*^ ^*∗*^ ^*∗*^*P* < 0.0001 vs. the control group; ^#^*P* < 0.05, ^####^*P* < 0.0001 vs. the diabetic group; and ^+++^*P* < 0.001 vs. the D-DM_E_. D-CM_E_, diabetic group receiving early WJMSCs-CM; D-CM_L_, diabetic group receiving late WJMSCs-CM; D-INS_E_, diabetic group receiving early insulin; D-INS_L_, diabetic group receiving late insulin; D-DM_E_, diabetic group receiving early DMEM; D-DM_L_, diabetic group receiving late DMEM; early, treatment was started immediately after diabetes induction; late, treatment was started 30 days after diabetes induction.

The body weight gain, testis weight, and gonadosomatic index of rats in all experimental groups are shown in Figures 3(a), 3(b), and 3(c). A significant weight loss was observed in the diabetic group. Early treatment with WJMSCs-CM or insulin and only late treatment with insulin significantly increased body weight gain. The testis weight was also reduced in diabetic rats compared to the control group. Although both early and late treatments with WJMSCs-CM and insulin increased testis weight in diabetic rats, it was only significant in D-INS_E_ compared to the untreated diabetic group. There were no differences in the gonadosomatic index among all groups. The early and late DMEM-treated diabetic rats did not differ in body weight, testis weight, or gonadosomatic index with the diabetes group. Body weight gain in the D-INS_E_ was higher than in the D-DM_E_. There were no differences in three parameters between late and early administration of WJMSCs-CM and insulin.

### 3.3. Serum FSH, LH, and Testosterone Levels

**Figure 4:**
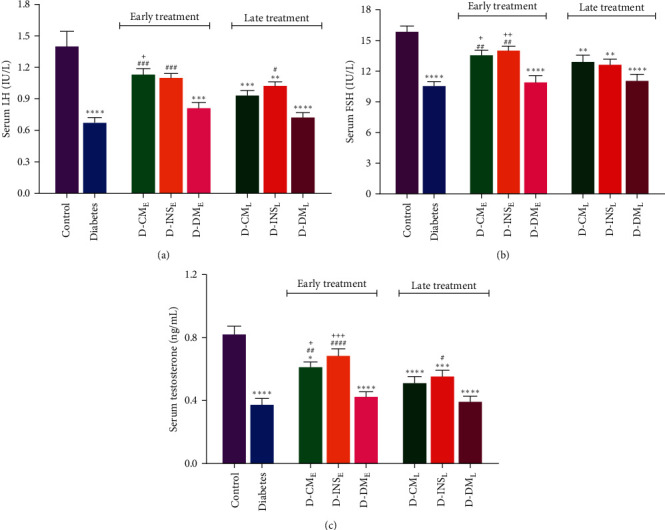
Serum LH (a), FSH (b), and testosterone (c), levels in the control, diabetes, early and late insulin, CM, and DMEM-treated diabetic groups. Data were analyzed by one-way ANOVA (Tukey's HSD test) and presented as mean ± SEM (*n* = 7).  ^*∗*^*P* < 0.05,  ^*∗∗*^*P* < 0.01,  ^*∗*^ ^*∗*^ ^*∗*^*P* < 0.001,  ^*∗*^ ^*∗*^ ^*∗*^ ^*∗*^*P* < 0.0001 vs. the control group; ^#^*P* < 0.05, ^##^*P* < 0.01, ^###^*P* < 0.001, ^####^*P* < 0.0001 vs. the diabetic group; and ^+^*P* < 0.05, ^++^*P* < 0.01, ^+++^*P* < 0.001 vs. the D-DM_E_ group. D-CM_E_, diabetic group receiving early WJMSCs-CM; D-CM_E_, diabetic group receiving early WJMSCs-CM; D-CM_L_, diabetic group receiving late WJMSCs-CM; D-INS_E_, diabetic group receiving early insulin; D-INS_L_, diabetic group receiving late insulin; D-DM_E_, diabetic group receiving early DMEM; D-DM_L_, diabetic group receiving late DMEM; early, treatment was started immediately after diabetes induction; Late, treatment was started 30 days after diabetes induction.

Figures 4(a), 4(b), and 4(c) illustrate the serum concentrations of LH, FSH, and testosterone. Compared to the control group, the serum LH, FSH, and testosterone levels were significantly lower in diabetic rats (*P*  < 0.0001). Early treatment with WJMSCs-CM and insulin led to a statistically significant increase in serum LH, FSH, and testosterone concentrations compared to diabetic rats. No statistically significant increment in serum levels of three hormones was observed in late WJMSCs-CM-treated rats. There were significant differences in serum levels of LH and testosterone between D-INS_L_ and diabetic groups. Early and late administration of DMEM did not change FSH, LH, and testosterone concentrations compared to the diabetes group. The serum levels of all hormones in the D-CM_E_ group and FSH and testosterone in the D-INS_E_ group were higher than in the D-DM_E_. There were no differences in sex hormones between early and late WJMSCs-CM and insulin administration.

### 3.4. Sperm Parameters

**Figure 5:**
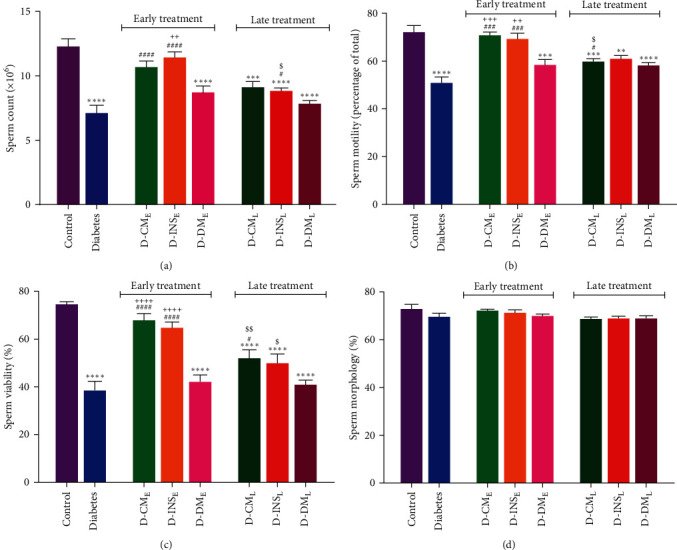
Count (a), motility (b), viability (c), and morphology (d) of sperm in the control, diabetes, early and late insulin, CM, and DMEM-treated diabetic groups. Data were analyzed by one-way ANOVA (Tukey's HSD test) and presented as mean ± SEM (*n* = 7).  ^*∗*^ ^*∗*^*P* < 0.01,  ^*∗*^ ^*∗*^ ^*∗*^*P* < 0.001,  ^*∗*^ ^*∗*^ ^*∗*^ ^*∗*^*P* < 0.0001 vs. the control group; ^#^*P* < 0.05, ^###^*P* < 0.001, ^####^*P* < 0.0001 vs. diabetic group; ^$^*P* < 0.05, ^$$^*P* < 0.01 vs. the early-treated diabetic group; and ^++^*P* < 0.01, ^+++^*P* < 0.001, ^++++^*P* < 0.0001 vs. the D-DM_E_ group. D-CM_E_, diabetic group receiving early WJMSCs-CM; D-CM_L_, diabetic group receiving late WJMSCs-CM; D-INS_E_, diabetic group receiving early insulin; D-INS_L_, diabetic group receiving late insulin; D-DM_E_, diabetic group receiving early DMEM; D-DM_L_, diabetic group receiving late DMEM; early, treatment was started immediately after diabetes induction; late, treatment was started 30 days after diabetes induction.

Statistical analysis of results showed that the total count (Figure 5(a)), motility (Figure 5(b)), viability (Figure 5(c)), and morphology (Figure 5(d)) of sperm were significantly lower in diabetic rats compared to the control group. Early treatment with WJMSCs-CM and insulin resulted in a statistically significant increase in three sperm parameters compared to the diabetic group. Although sperm count in the D-INS_L_ group and sperm motility and viability were significantly increased in the D-CM_L_ group, they did not reach the control group. Statistically significant differences in these sperm parameters were observed between D-INS_L_ and control groups. There were significant differences in sperm motility and viability between D-CM_E_ and D-CM_L_, as well as between D-INS_E_ and D-INS_L_ in sperm viability. No differences in sperm parameters were found between early and late DMEM-treated diabetic rats and the diabetes group. Compared to the D-DM_E_ group, the D-INS_E_ group showed significant differences in sperm count, motility, and viability. In contrast, significant differences in sperm motility and viability were observed in the D-CM_E_ group compared to the D-DM_E_ group. There were no differences in sperm morphology among all experimental groups (Figure 5(d)).

### 3.5. Testicular Oxidative Stress Parameters

**Figure 6:**
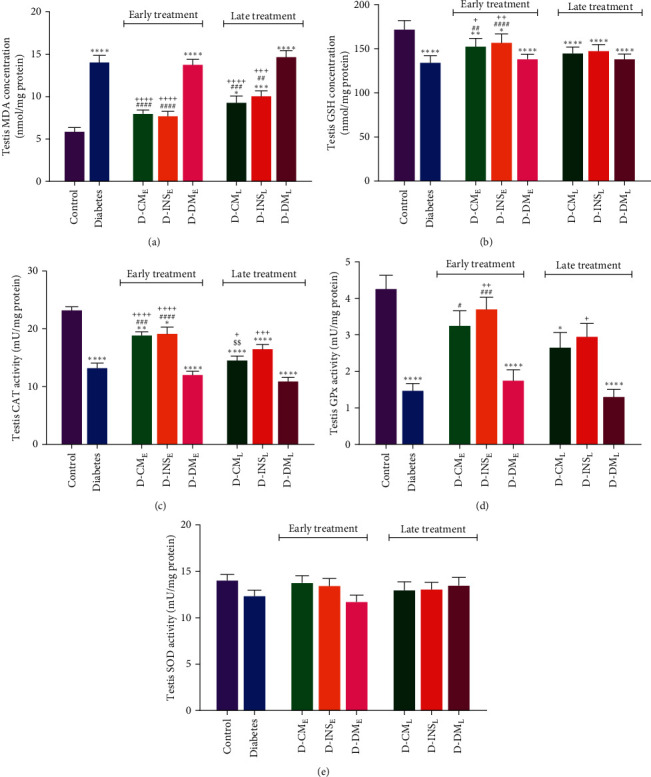
MDA (a), GSH (b) levels and specific activity of catalase (c), GPx (d), and SOD (e) of testis in the control, diabetes, early and late insulin, CM, and DMEM-treated diabetic groups. Data were analyzed by one-way ANOVA (Tukey's HSD test) and presented as mean ± SEM (*n* = 7).  ^*∗*^*P* < 0.05,  ^*∗*^ ^*∗*^P < 0.01,  ^*∗*^ ^*∗*^ ^*∗*^P < 0.001,  ^*∗*^ ^*∗*^ ^*∗*^ ^*∗*^*P* < 0.0001 vs. the control group; ^#^*P* < 0.05, ^##^*P* < 0.01, ^###^*P* < 0.001, ^####^*P* < 0.0001 vs. the diabetic group; ^$$^*P* < 0.01 vs. the early-treated diabetic group; ^+^*P* < 0.05, ^++^*P* < 0.01, ^+++^*P* < 0.001, ^++++^*P* < 0.0001 vs. the D-DM_E_ and D-DM_L_ groups. D-CM_E_, diabetic group receiving early WJMSCs-CM; D-CM_L_, diabetic group receiving late WJMSCs-CM; D-INS_E_, diabetic group receiving early insulin; D-INS_L_, diabetic group receiving late insulin; D-DM_E_, diabetic group receiving early DMEM; D-DM_L_, diabetic group receiving late DMEM; early, treatment was started immediately after diabetes induction; late, treatment was started 30 days after diabetes induction.

Figures 6(a), 6(b), 6(c), 6(d), and 6(e) present testicular MDA and GSH levels and specific activity of SOD, GPx, and catalase in all of the experimental groups. The results of this study showed that the MDA level (Figure 6(a)) of testicular tissue in the diabetic group was significantly (*P*  < 0.05) higher than that in the control group. The GSH concentration (Figure 6(b)) and specific activity of antioxidant enzymes of catalase (Figure 6(c)) and GPx (Figure 6(d)) were reduced in the diabetic group compared to the control group. Early and late treatments of WJMSCs-CM and insulin resulted in a significant decrease in the testicular MDA level compared to the control group. However, they did not reach the control group in both D-CM_L_ and D-INS_L_ groups. Early- and late-treated rats with WJMSCs-CM and insulin showed significant differences in testicular MDA concentrations compared to early and late DMEM-treated rats. There were no significant differences in testicular MDA concentrations in early- and late-treated rats with WJMSCs-CM and insulin.

After early treatment of diabetic rats with WJMSCs-CM and insulin, the testicular GSH concentration was significantly raised, but it was still considerably lower than the control group. Compared to diabetic rats, late treatment with WJMSCs-CM and insulin did not alter testicular GSH levels in D-CM_L_ and D-INS_L_ groups (Figure 6(b)). Only early-treated rats with WJMSCs-CM and insulin showed a significant difference in testicular GSH level compared to early DMEM-treated rats. There was no significant difference in testicular GSH concentration in early- and late-treated rats with WJMSCs-CM and insulin.

A significant increase in catalase (CAT) and GPx activity was observed in the diabetic group after early treatment with WJMSCs-CM and insulin. However, catalase activity did not reach the levels of the control group (Figures 6(c) and 6(d)). Compared to diabetic rats, there was no significant alteration in testicular catalase and GPx activity in the D-CM_L_ and D-INS_L_ groups. Early- and late-treated rats with WJMSCs-CM and insulin showed substantial differences in testicular catalase activity compared to early and late DMEM-treated rats. The testicular GPx activity in early and late insulin-treated rats was higher than in early and late DMEM-treated rats. Early and late administration of DMEM did not change oxidative stress parameters in the diabetes group. There was a significant difference in testicular catalase activity in early- and late-treated rats with WJMSCs-CM. There was no significant difference in testicular SOD activity among all groups (Figure 6(e)).

### 3.6. Testicular Bax, Bcl-2, and TNF-*ɑ* Gene Expression

**Figure 7:**
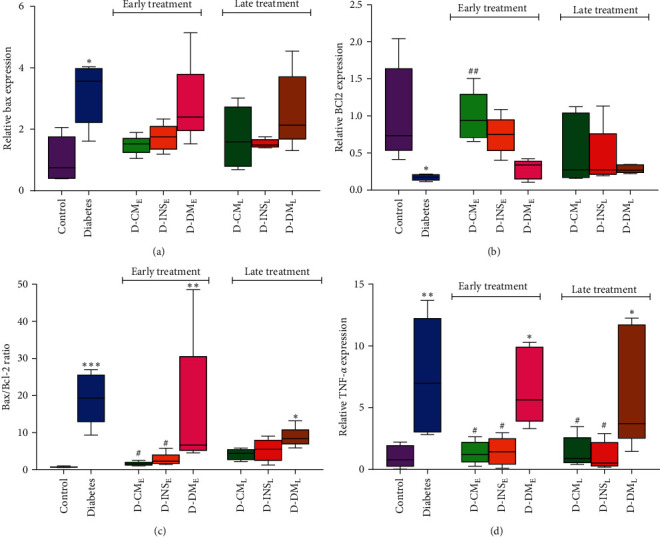
Changes in the testicular Bcl-2 (a), Bax (b), Bax/Bcl-2 ratio (c), and TNF-*ɑ* (d) gene expression in the control, diabetes, early and late insulin, CM, and DMEM-treated diabetic groups. The BAX, Bcl2, and Bax/Bcl2 data were statistically analyzed using the Kruskal–Wallis nonparametric test and reported as median with IQR. The variable of TNF-*ɑ* was analyzed by one-way ANOVA (Tukey's HSD test) and presented as mean ± SEM (*n* = 7).  ^*∗*^*P* < 0.05,  ^*∗*^ ^*∗*^*P* < 0.01,  ^*∗*^ ^*∗*^ ^*∗*^*P* < 0.001 vs. the control group; ^#^*P* < 0.05, ^##^*P* < 0.01 vs. the diabetic group. D-CM_E_, diabetic group receiving early WJMSCs-CM; D-CM_L_, diabetic group receiving late WJMSCs-CM; D-INS_E_, diabetic group receiving early insulin; D-INS_L_, diabetic group receiving late insulin; D-DM_E_, diabetic group receiving early DMEM; D-DM_L_, diabetic group receiving late DMEM; early, treatment was started immediately after diabetes induction; late, treatment was started 30 days after diabetes induction.

The results depicted in Figures 7(a) and 7(b) demonstrate that the gene expression levels of Bax and Bcl-2 in testis were significantly elevated and reduced, respectively, in the untreated diabetes group compared to the control group (*P*  < 0.01). Additionally, the testicular Bax/Bcl-2 ratio (Figure 7(c)) was significantly higher (*P*  < 0.01) in the diabetes group compared to the control group. Although early and late treatments with WJMSCs-CM and insulin decreased the level of Bax expression in diabetic rats, the reduction was not significant (Figure 6(a)). On the other hand, the mRNA expression level of Bcl-2 in D-CM_E_ (*P*  < 0.05), not in D-CM_L_, was significantly higher than that in the diabetic group. There was no significant increase in Bcl-2 expression level in D-INS_E_ and D-INS_L_ compared to diabetic groups (Figure 6(b)). The testicular Bax/Bcl-2 ratio was decreased in diabetic groups with early WJMSCs-CM and insulin treatment compared to the untreated diabetic group. Although in comparison diabetic group, late treatment with WJMSCs-CM and insulin reduced the testicular Bax/Bcl-2 ratio in D-CM_L_ and D-INS_L_ groups, this reduction was not significant (Figure 6(c)).

Also, the mRNA expression level of TNF-*ɑ* in the diabetic group was significantly increased compared to the control group (*P*  < 0.01). However, early and late treatments with WJMSCs-CM and insulin significantly decreased the expression level of TNF-*ɑ* mRNA compared to the untreated diabetes group (Figure 6(d)).

There were no significant differences in the gene expression levels of Bax, Bcl-2, and TNF-*ɑ* in early- and late-treated rats with WJMSCs-CM and insulin. Compared to the diabetes group, early and late DMEM administrations did not affect the gene expression levels of Bax, Bcl-2, and TNF-*ɑ*.

### 3.7. Histological Examination of the Testes

**Figure 8:**
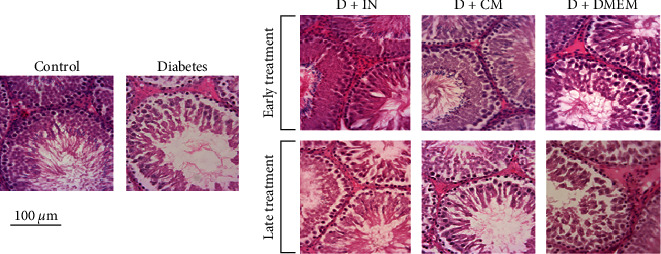
The microscopic images of seminiferous tubules of rat testis tissue in the control, diabetes, early and late insulin, WJMSCs-CM, and DMEM-treated diabetic groups. Normal appearance of spermatogenic, Leydig, and Sertoli cells was observed in the seminiferous tubules of control rats. Loss of spermatogenic cell populations in the epithelium, reduction of spermatozoa in the tubule lumen, and thinning of the seminiferous epithelium could be observed in the diabetic group. The histological changes in the epithelium and lumen of testis seminiferous tubules in the D-CM_E_ group and especially in the D-INS_E_ group appear to be similar to normal histology. Photomicrograph of testicular tissue in late treated rats with insulin, WJMSCs-CM showed little disorganized seminiferous tubules lined by few layers of spermatogenic cells. The hematoxylin & eosin (H&E) stained testis sections. The scale bar is 100 *μ*m. D-CM_E_, diabetic group receiving early WJMSCs-CM; D-CM_L_, diabetic group receiving late WJMSCs-CM; D-INS_E_, diabetic group receiving early insulin; D-INS_L_, diabetic group receiving late insulin; D-DM_E_, diabetic group receiving early DMEM; D-DM_L_, diabetic group receiving late DMEM; early, treatment was started immediately after diabetes induction; late, treatment was started 30 days after diabetes induction.

Results obtained from light microscopic examination of H&E-stained right testicular sections of all rats indicated that the seminiferous tubules in the control groups exhibited typical morphology of spermatogenic, Leydig, and Sertoli cells. In the diabetic group, atrophy of seminiferous tubules, loss of spermatogenic cells in the epithelium, decreased spermatozoa in the tubule lumen, and thinning of the seminiferous epithelium were observed. On the contrary, samples from early WJMSCs-CM and insulin-treated rats showed normal seminiferous tubules in both groups of D-CM_E_ and D-INS_E_. It appears that early treatment with WJMSCs-CM and insulin could prevent seminiferous tubule volume and spermatogenic cell loss. Late treatment with WJMSCs-CM and insulin slightly ameliorated diabetes-induced histological changes in the testis. A few irregular spermatogenic tubules covered with a few layers of spermatogenic cells were still observed in the D-CM_L_ and D-INS_L_ groups. Like the untreated diabetes group, early and late DMEM-treated groups indicated marked tubular disorganization and a few layers of spermatogenic cells (Figure 8).

### 3.8. Stereological Analyses of the Testes

#### 3.8.1. Volume Estimation

**Figure 9:**
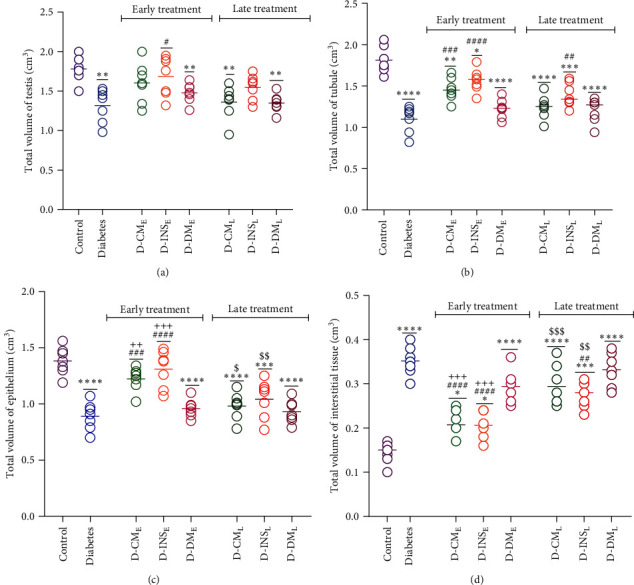
Effects of WJMSCs-CM and insulin on total volumes of the testis (a), seminiferous tubules (b), seminiferous epithelium (c), and interstitial tissue (d). Data were analyzed by one-way ANOVA (Tukey's HSD test) and presented as mean ± SEM (*n* = 7).  ^*∗*^*P* < 0.05,  ^*∗*^ ^*∗*^*P* < 0.01,  ^*∗*^ ^*∗*^ ^*∗*^*P* < 0.001,  ^*∗*^ ^*∗*^ ^*∗*^ ^*∗*^*P* < 0.0001 vs. the control group; ^#^*P* < 0.05, ^##^*P* < 0.01, ^###^*P* < 0.001, ^####^*P* < 0.0001 vs. the diabetic group; ^$^*P* < 0.05, ^$$^*P* < 0.01, ^$$$^*P* < 0.001 vs. the early-treated diabetic group; and ^++^*P* < 0.01, ^+++^*P* < 0.001 vs. the D-DM_E_. D-CM_E_, diabetic group receiving early WJMSCs-CM; D-CM_L_, diabetic group receiving late WJMSCs-CM; D-INS_E_, diabetic group receiving early insulin; D-INS_L_, diabetic group receiving late insulin; D-DM_E_, diabetic group receiving early DMEM; D-DM_L_, diabetic group receiving late DMEM; early, treatment was started immediately after diabetes induction; late, treatment was started 30 days after diabetes induction.

As shown in Figures 9(a), 9(b), and 9(c), the total volumes of the testis, seminiferous tubules, and seminiferous epithelium in the diabetes group were significantly decreased compared to the control group. Contrarily, the total volume of interstitial tissue was increased in the diabetes group. Early treatment with WJMSCs-CM and insulin increased the total volumes of the testis in both D-CM_E_ and D-INS_E_ groups, which was only significant in D-INS_E_ (*P*  < 0.05). Although the total volumes of seminiferous tubules in the D-CM_E_ and D-INS_E_ groups were significantly higher than those in the diabetic group, they did not reach control. Early treatment with WJMSCs-CM and insulin increased epithelium volume and decreased interstitial tissue volume in the D-CM_E_ and D-INS_E_ groups (*P* < 0.0001). Late treatment with WJMSCs-CM does not affect all the above parameters. In contrast, late treatment with insulin partially improved the total volumes of seminiferous tubules and interstitial tissue in the D-INS_L_ group. No significant differences existed in the total volumes of the testis, seminiferous tubules, epithelium, and interstitial tissue between the early and late DMEM-treated groups and the diabetic group. Only early-treated rats with WJMSCs-CM and insulin showed substantial differences in epithelium and interstitial tissue volumes compared to early DMEM-treated rats. Significant differences existed in the total epithelium and interstitial tissue volumes between early- and late-treated rats with WJMSCs-CM and insulin.

#### 3.8.2. The Length Assessment of Seminiferous Tubules

**Figure 10:**
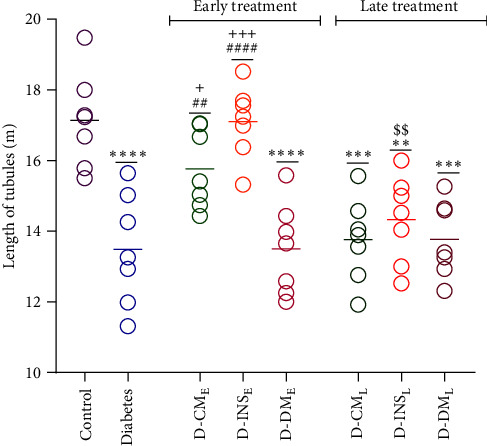
Effects of WJMSCs-CM and insulin on length of seminiferous tubules. Data were analyzed by one-way ANOVA (Tukey's HSD test) and presented as mean ± SEM (*n* = 7).  ^ ^*∗*^ ^*∗*^^*P* < 0.01,  ^ ^*∗*^ ^*∗*^ ^*∗*^^*P* < 0.001,  ^ ^*∗*^ ^*∗*^ ^*∗*^ ^*∗*^^*P* < 0.0001 vs. the control group; ^##^*P* < 0.01, ^####^*P* < 0.0001 vs. the diabetic group; ^$$^*P* < 0.01 vs. the early-treated diabetic group; and ^+^*P* < 0.05, ^+++^*P* < 0.001 vs. the D-DM_E_. D-CM_E_, diabetic group receiving early WJMSCs-CM; D-CM_L_, diabetic group receiving late WJMSCs-CM; D-INS_E_, diabetic group receiving early insulin; D-INS_L_, diabetic group receiving late insulin; D-DM_E_, diabetic group receiving early DMEM; D-DM_L_, diabetic group receiving late DMEM; early, treatment was started immediately after diabetes induction; late, treatment was started 30 days after diabetes induction.

The length of seminiferous tubules in all experimental groups is shown in Figure 10. Diabetes caused a reduction in the length of tubules compared to control. Early treatment with WJMSCs-CM and insulin led to a significant increase in the length of tubules in both D-CM_E_ and D-INS_E_ groups in comparison to the diabetic group. However, no difference in this parameter was observed between the late WJMSCs-CM and insulin-treated diabetic groups and the untreated diabetic group. There was no significant difference in the length of seminiferous tubules between the early and late DMEM-treated groups and the diabetic group. The length of seminiferous tubules in the D-CM_E_ and D-INS_E_ groups was higher than in the D-CM_E_ group. Only a significant difference existed in the seminiferous tubule length between early- and late-treated rats with insulin.

#### 3.8.3. Number of Cells

**Figure 11:**
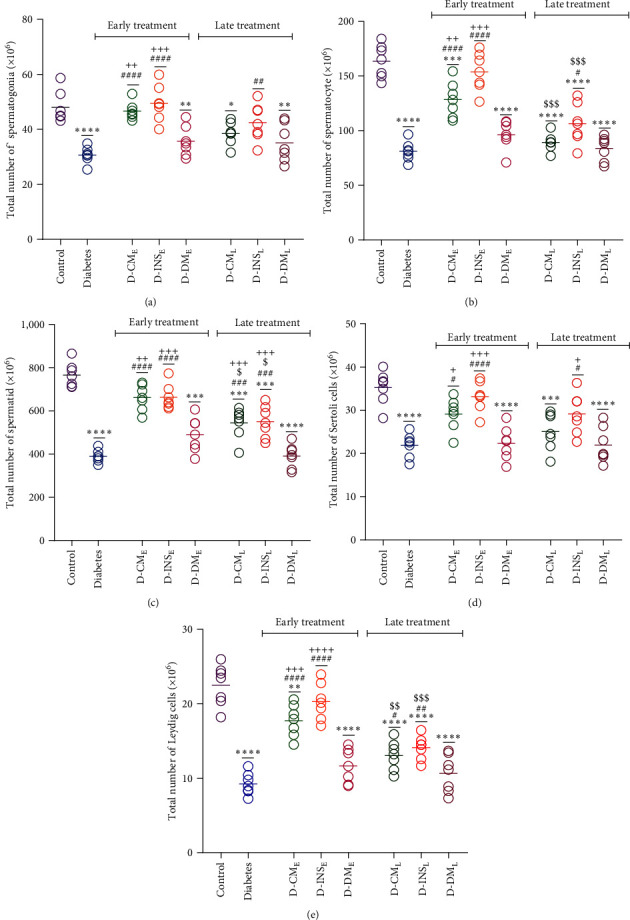
Effects of WJMSCs-CM and insulin on numbers of spermatogonia (a), spermatocyte (b), spermatid (c), Sertoli (d), and Leydig (e) cells of the testis. Data were analyzed by one-way ANOVA (Tukey's HSD test) and presented as mean ± SEM (*n* = 7). ^*∗*^*P* < 0.05,  ^*∗*^ ^*∗*^*P* < 0.01,  ^*∗*^ ^*∗*^ ^*∗*^*P* < 0.001,  ^*∗*^ ^*∗*^ ^*∗*^ ^*∗*^*P* < 0.0001 vs. the control group; ^#^*P* < 0.05, ^##^*P* < 0.01, ^###^*P* < 0.001, ^####^*P* < 0.0001 vs. the diabetic group; ^$^*P* < 0.05, ^$$^*P* < 0.01, ^$$$^*P* < 0.001 vs. the early-treated diabetic group; and ^+^*P* < 0.05, ^++^*P* < 0.01, ^+++^*P* < 0.001, ^++++^*P* < 0.0001 vs. the D-DM_E_. D-CM_E_, diabetic group receiving early WJMSCs-CM; D-CM_L_, diabetic group receiving late WJMSCs-CM; D-INS_E_, diabetic group receiving early insulin; D-INS_L_, diabetic group receiving late insulin; D-DM_E_, diabetic group receiving early DMEM; D-DM_L_, diabetic group receiving late DMEM; early, treatment was started immediately after diabetes induction; late, treatment was started 30 days after diabetes induction.

The total number of spermatogenic cells, Sertoli, and Leydig cells was reduced in the testes of diabetic rats in comparison to the control group ((Figures 11(a), 11(b), 11(c), 11(d), and 11(e)). Early treatment with WJMSCs-CM and insulin significantly increased the total number of all the above cells in the D-CM_E_ and D-INS_E_ compared to the diabetic group so that the total number of spermatogonia, spermatid, and Sertoli in early WJMSCs-CM-treated diabetic groups, and all above cells in early insulin-treated diabetic groups reach control. In comparison to the diabetic group, a remarkable increase in the total number of spermatogenic cells, Sertoli, and Leydig cells was also observed in the D-INS_L_ group in which only the total number of spermatogonia and Sertoli was near to control. There were also significant differences in the total number of spermatid and Leydig cells between the D-CM_L_ and diabetic groups, but they did not reach the control group. Early and late treatments with DMEM did not affect all the above parameters in diabetic groups. Two groups D-CM_E_ and D-INS_E_ showed significant differences in all spermatogenic cells, Sertoli, and Leydig cells compared to D-DM_E_. The total number of spermatid and Sertoli cells in the D-INS_L_ and only the number of spermatid cells in the D-CM_L_ were higher than in the D-DM_L_. The number of spermatocytes, spermatid, and Leydig cells differed between early and late groups treated with WJMSCs-CM and insulin.

## 4. Discussion

Diabetes induces testicular injury and abnormalities in sperm. MSCs secretion-based CM has a repair function similar to MSCs but without tumor growth [34, 35]. In the present study, we investigated the early and late treatments of WJMSCs-CM on male reproductive health in the STZ-induced rat model of diabetes.

Our study found that STZ-induced diabetes leads to higher fasting blood glucose and lower serum insulin levels by destroying pancreatic islet cells through oxidative stress, metabolic disruption, and impaired mitochondrial function [36]. However, early and late administration of WJMSCs-CM to diabetic animals reduced hyperglycemia and enhanced insulin production. Previous research suggests that stem cell-derived CM regenerates pancreatic *β* cells for insulin production [18, 37].

In line with several studies, the present investigation demonstrated that STZ-induced diabetes lowered the body and testis weights in rats, likely due to protein wasting from insulin deficiency [38, 39, 40]. Both early and late insulin administration significantly increased body and testis weights, while MSCs-CM demonstrated partial improvements.

Our study showed that STZ-induced diabetes reduces male sex hormone levels, sperm count, motility, and viability, aligning with previous research [41, 42, 43, 44]. It is well-known that FSH and LH promote germ cell maturation and function in the gonads. Inadequate insulin in diabetes damages the HPG axis, decreasing GnRH, FSH, LH, and testosterone, leading to testicular atrophy, seminiferous tubules damage, and spermatogenic cell impairment [2]. In the current study, diabetic rats showed significantly lower serum testosterone levels, likely due to reduced insulin stimulation of Leydig cells and hyperglycemia-induced oxidative damage to these cells [2, 45]. Testosterone promotes protein synthesis in spermatogenic cells and reduced testosterone impairs protein synthesis in germ cells, causing germ cell degeneration, abnormalities in spermatogenesis, and low sperm count [46, 47].

MSCs primarily exert their therapeutic effects via paracrine signaling of their secretome, which includes growth factors, cytokines, chemokines, enzymes, and extracellular vesicles like exosomes and microvesicles [48, 49]. Important growth factors of WJMSCs-CM are hepatocyte growth factor (HGF), fibroblast growth factor-2 (FGF2), angiogenesis factors like vascular endothelial growth factor (VEGF), nerve growth factor (NGF), brain-derived neurotrophic factor (BDNF), and transforming growth factor *β* (TGF*β*) [17, 50]. Some receptors for these growth factors are found in spermatozoa, indicating their role in improving sperm quality and male reproductive health [51]. Sharifian et al. [52] showed that CM derived from MSCs can improve sperm count and motility in the ischemia/reperfusion rat model. Several studies have also documented the important role of some secreted growth factors of CM, such as NGF, VEGF, IGF, and TGF*β*, in the regulation of spermatogenesis, germ cell maturation, spermatozoa survival, steroidogenesis stimulation, and HPG axis activation [51, 53, 54].

The testis is vulnerable to oxidative stress due to its high concentration of unsaturated fatty acids and ROS-generating systems [55]. Hyperglycemia can adversely affect testicular tissue, including oxidative stress, inflammation, and apoptosis [8, 56]. The level of MDA in tissues is a crucial diagnostic indicator of oxidative stress, which can cause apoptosis through intra- and intermolecular cross-linkages. Increased ROS leads to lipid peroxidation in cell membranes, impacting the high mitotic activity of spermatogenic cells [57]. Studies have shown a reduction in antioxidative enzyme expression and an increase in the levels of MDA in the testis of rats with STZ-induced diabetes [8, 58, 59]. Our investigation revealed that diabetes dramatically enhanced MDA levels and decreased GSH concentration and the antioxidant enzyme activity of GPx and catalase. Additionally, hyperglycemia-induced oxidative stress triggers a pro-inflammatory and apoptotic signaling pathway, with ROS initiating inflammation [60, 61]. ROS stimulates the synthesis and release of various pro-inflammatory molecules, including leukotrienes and cytokines [62]. TNF-*α* is one of the important pro-inflammatory cytokines. Previous studies have indicated an increase in TNF-*α* expression in the testes of diabetic rats [56]. This research found that untreated diabetic rats showed inflammation in their testes, leading to a significant increase in TNF-*α* mRNA expression, triggering inflammatory, and apoptotic responses [56, 63].

The present study found that early and late administration of WJMSCs-CM and insulin reduced testicular lipid peroxidation and increased antioxidant enzyme activity in diabetic rats. Researchers discovered that major growth factors of conditioned media, such as HGF, FGF2, VEGF, NGF, and BDNF, have antioxidant properties and lower ROS in various tissues [51, 64, 65]. WJMSCs-CM and insulin also demonstrated anti-inflammatory actions, reducing TNF-*α* mRNA expression levels in diabetic rats' testis. Studies have indicated that the conditioned media derived from MSCs have anti-inflammatory properties, reducing the expression of pro-inflammatory molecules, such as TNF-*α*, IL-1, and IL-6 in various tissues [18, 66].

The present study found that early and late administration of WJMSCs-CM and insulin reduced testicular lipid peroxidation and increased antioxidant enzyme activity in diabetic rats. Researchers discovered that major growth factors of conditioned media, such as HGF, FGF2, VEGF, NGF, and BDNF, have antioxidant properties and lower ROS in various tissues.

On the other hand, apoptotic cell death is crucial for testicular injury in diabetic rats [67]. Consistent with previous research [56, 68], our study found a significant increase in mRNA expression of pro-apoptotic factor Bax and a decrease in antiapoptotic factor Bcl2 in the testis of untreated diabetic rats. The ratio of the apoptotic protein Bax to the antiapoptotic protein Bcl-2, which triggers the caspase cleavage cascade, is a critical factor in determining apoptosis [69]. Our findings indicated that early treatment with conditioned media derived by MSCs significantly decreased the Bax/Bcl2 ratio in the testicular tissue of MSCs-CM-treated diabetic rats. MSCs-CM contains secretory factors, including chemokines, cytokines, growth factors, lipids, and free nucleic acids that regulate cellular processes like proliferation, differentiation, immunomodulation, antiapoptotic mechanisms, and anti-inflammatory responses [35, 47]. Studies have shown that growth factors like HGF, BDNF, NGF, and VEGF, present in MSCs-CM, can protect sperm from apoptosis, improve sperm motility and viability, and promote testes recovery by upregulating mRNA and protein expression [47, 51].

This study investigates the impact of diabetes on the testes' histological structure. Consistent with numerous investigations [2, 70, 71], our research showed an increase in interstitial tissue, a decrease in the weight and volume of testes, and a reduction in seminiferous tubule length and volume in diabetic rats. It was found that the length and volume of the tubules are related to the height of the germinal epithelium [72]. Our finding revealed that, in diabetic rats, a reduction in volume seminiferous tubule volume was accompanied by a decrease in germinal epithelium volume, indicating that seminiferous tubules atrophied as diabetes progressed. Diabetes also negatively affects the population of spermatogonia, spermatocytes, spermatids, and Sertoli cells, reducing germinal epithelium volume. A study showed that MSCs-CM can reduce histological changes caused by ischemia and reperfusion [52]. Our study showed that treatment with WJMSCs-CM significantly improved the structural defects in the testis of diabetes rats. This improvement can be attributed to secreted substances by stem cells like growth factors, cytokines, and protective proteins.

## 5. Conclusion

Our study showed the therapeutic potential of WJMSC_S_-CM in treating testicular injury and sperm abnormalities caused by diabetes. The anti-inflammatory, antioxidant, and regenerative properties of MSC-derived secretome can effectively reduce diabetes's negative impact on reproductive health. It is noteworthy that growth factors, exosomes/microvesicles, and other biologically active substances of WJMSCs-CM can potentially cause these effects, and one of the limitations of our study was the lack of clarity about the specific bioactive molecules and their amount in the CM that mediate the therapeutic effects on testicular dysfunction caused by diabetes. Future research should focus on identifying these bioactive substances and their molecular mechanisms. In addition, to find the best treatment strategy and optimize its use, it is essential to test various dosages, frequencies, and durations of CM administration. It is recommended that additional studies be done on the combination of SMCs-CM with other methods of therapy for diabetes reproductive disorder.

Overall, the outcomes of our research can provide a basis for future clinical trials and the utilization of WJMSC_S_-CM as a therapeutic option. This study also demonstrated that early treatment with WJMSCs-CM was more effective than late treatment in ameliorating diabetic male reproductive dysfunction.

## Data Availability

All data used to support the findings of this study were analyzed and included in the article.
